# Supplementary material for: Early joint attention abilities measured by the ADOS‐2 predict subsequent expressive language development in minimally verbal autistic children
Source: JCPP Adv. 2026 Jun 11:e70140. Online ahead of print. doi: 10.1002/jcv2.70140 (PMC13339333; doi:10.1002/jcv2.70140)
Supplement: Supplementary file 1 — Table S1 [file JCV2-9999-e70140-s001.docx]

**Early joint attention abilities measured by the ADOS-2 predict subsequent expressive language development in minimally verbal autistic children**

**Supporting Information**

**Table S1:** **Imputation of Missing Data**

In the current sample 30.3% of the children did not complete the Bayley-III assessment at T1 and we chose to impute their data (see Methods). To ensure that imputation of missing data did not introduce systematic bias (e.g., that we only analyzed "higher-functioning" children), we performed a sensitivity analysis where we compared all baseline measures across the children who completed cognitive versus those who did not using t-tests. The results below indicate that there were no systematic differences across the two groups, indicating that the missing data was randomly distributed in children with variable baseline characteristics.

| **Variable** | **p-value** | **Statistical Method** | **Status** |
| --- | --- | --- | --- |
| Age of diagnosis (months) | 0.502 | *t*-test | No Bias (OK) |
| ADOS-2 SA CSS T1 | 0.340 | *t*-test | No Bias (OK) |
| ADOS-2 RRB CSS T1 | 0.427 | *t*-test | No Bias (OK) |
| ADOS-2 total CSS T1 | 0.576 | *t*-test | No Bias (OK) |
| ADOS-2 JA T1 | 0.154 | *t*-test | No Bias (OK) |
| ADOS-2 Language T1 | 0.676 | *t*-test | No Bias (OK) |
| Maternal years of education | 0.165 | *t*-test | No Bias (OK) |
| Paternal years of education | 0.085 | *t*-test | No Bias (OK) |
| Duration of special education | 0.533 | *t*-test | No Bias (OK) |
| Father age at diagnosis | 0.450 | *t*-test | No Bias (OK) |
| Mother age at diagnosis | 0.462 | *t*-test | No Bias (OK) |
| Sex | 0.384 | Fisher’s Exact | No Bias (OK) |

SA - Social Affect; RRB - Restricted and Repetitive Behavior; ADOS-2 - Autism Diagnostic Observation Schedule, 2nd edition; JA – Joint Attention; CSS – Calibrated Severity Score. All *p*-values were non-significant (p > .05), indicating no systematic bias between groups.
